# Evaluating the understanding about kidney stones among adults in the United Arab Emirates

**DOI:** 10.1016/j.jtumed.2021.04.005

**Published:** 2021-05-25

**Authors:** Hussain S. Aldaher, Safa Z. Kadhim, Nora M. Al-Roub, Ahmed H. Alsadi, Dana A. Salam, Eva A. Tillo

**Affiliations:** University of Sharjah, College of Medicine, United Arab Emirates

**Keywords:** علم الأوبئة, المعرفة, حصى الكلى, حصوات الكلى, عوامل الخطر., Kidney stones, Knowledge, Prevalence, Renal stones, Risk factors

## Abstract

**Objectives:**

The prevalence of kidney stones is increasing worldwide. Multiple risk factors are believed to contribute to the development of kidney stones such as lifestyle, diet, and global warming. In the United Arab Emirates (UAE), there has been limited research exploring the prevalence and risk factors of kidney stones. This study attempts to assess the understanding and prevalence of kidney stones among adults in the UAE.

**Methods:**

In this cross-sectional study, data were collected using a self-administered questionnaire, distributed among 515 participants (20–49 years old) from Abu Dhabi, Dubai, Ajman, and Sharjah states. IBM SPSS version 25 was used for data analysis.

**Results:**

The mean of knowledge score was 56.4% (n = 500). There was no correlation between the knowledge of those who had experienced kidney stones and those who did not. Furthermore, a family history of kidney stones increased the risk of developing stones by 2.27 times. Among participants reporting signs, symptoms, diagnosis, and the management of kidney stones, the knowledge and understanding about kidney stones was high. However, the perceptions of the same cohort about dietary precautions were limited. While analysing the sources of knowledge, the Internet and mass media were twice as important as physicians in educating the population.

**Conclusion:**

This study shows that the study cohort from the UAE population was aware of certain aspects of kidney stones but was quite naïve about its consequential risk factors. This highlights the importance of promoting education about kidney stones through health campaigns.

## الملخص

**أهداف البحث:**

تزايد انتشار حصى الكلى في جميع أنحاء العالم في العقد الماضي، ويُعتقد أن عوامل الخطر المتعددة تُعزى إلى التغيير في نمط الحياة، والنظام الغذائي، وحتى الاحتباس الحراري. في دولة الإمارات العربية المتحدة، لم يتم عمل الكثير من البحوث لاستكشاف انتشار حصى الكلى والمعرفة عنها. تهدف هذه الدراسة إلى قياس مدى معرفة المجتمع بحصى الكلى بين البالغين في دولة الإمارات العربية المتحدة.

**طرق البحث:**

في هذه الدراسة المقطعية، تم جمع البيانات باستخدام استبانة ذاتية الإدارة تم توزيعها علىخمسة مائة و خمسة عشر مشارك (٢٠-٤٩ عاما) من أبو ظبي ودبي وعجمان والشارقة.

**النتائج:**

من بين ٥٠٠ مشارك، بلغ متوسط مجموع درجات المعرفة ٥٦.٤٪. لم يكن هناك ارتباط بين المعرفة بين أولئك الذين عانوا من حصى الكلى والذين لم يصابوا بها. كان وجود تاريخ عائلي بتشخيص حصى الكلى يزيد من خطر الإصابة بحصى الكلى بمعدل ٢.٢٧. وأظهرت النتائج أن المشاركين الذين كان لديهم علامات وأعراض وتشخيص وعلاج حصى الكلى، كان لديهم مستوى جيد من المعرفة بعد تحليل مصدر المعرفة، ولكن كان هناك مستوى ضئيل من المعرفة بخصوص العوامل الغذائية المتعلقة بحصى الكلى. كان للإنترنت ووسائل الإعلام دور أكبر بضعفين من الأطباء في تثقيف السكّان.

**الاستنتاجات:**

استطاعت هذه الدراسة أن توضح أن سكّان الإمارات على دراية ببعض الجوانب المتعلقة بحصوات الكلى ولكن على غير دراية بشأن عوامل الخطر التبعية. وهذا يسلط الضوء على أهمية تعزيز التعليم من خلال الحملات الصحية.

## Introduction

Up to 12% of the world's population will develop kidney stones at some stage in their lives.^1^ It is one of the most common urinary tract disorders. There are no symptoms when the stone initially forms, but it can later present as severe flank pain, haematuria (blood in urine), urinary tract infection, blockage of urine flow, and hydronephrosis (dilation of kidney).^2^ Kidney stones increase the risk of developing chronic kidney disease by 60% and end-stage renal disease by 40%,^3^ it has been also associated with the development of papillary renal cell carcinoma (RCC).^4^

Kidney stones are among the most painful urological disorders. Recently, it has become highly prevalent worldwide (7–13% in North America, 5–9% in Europe, 1–5% in Asia); its high occurrence rate and the expensive nature of disease management necessitates increasing awareness in the population^4^, ^5^, ^6^

Studies have confirmed the multi-factorial nature of the disease, and one of the most important determinants increasing the risk of developing stones is the exposure to high temperatures.^7^ The hot climate in the United Arab Emirates (UAE), especially in the summer, and inadequate compensatory water intake or re-hydration can dramatically influence the possibility of disease occurrence regardless of the age or gender.

Moreover, there is evidence that diet and food choice can seriously influence stone formation; however, the study revealed confusion in the choice of certain diets among the public.^8^ According to recent studies, the prevalence of obesity and associated non-communicable diseases like diabetes are 32.3% and 15.5%, respectively, among expatriates in the UAE (which is the predominant population); these diseases may continue to rise with lack of interventions,^9^ and subsequently increase the prevalence of kidney stones.

The importance of preventative strategies in managing the rising prevalence of kidney stone disease is severely underestimated. Guidelines and studies advocate the importance of dietary evaluation and counselling as part of the management of kidney stones, and adherence to these can drastically decrease the morbidity. However, such counselling is complex and the clinician is usually responsible to implement it effectively.^10^, ^11^, ^12^

The objective of this study is to determine the basic level of understanding about kidney stones and their risk factors, to investigate misconceptions, as well as to assess the potential sources of knowledge. This study seeks to address the highlight role of physicians in educating patients and spreading awareness in the population.^13^

## Materials and Methods

This is a quantitative cross-sectional study that focuses on determining public knowledge and awareness regarding renal stones in the UAE. Data were collected using self-administered questionnaires comprising 15 questions, from adults aged between 20 and 49 years old, from Abu-Dhabi, Dubai, Sharjah, and Ajman during October 2018. By.

### Sampling method

The target population consists of adults between the ages of 20–49 years in the UAE. The inclusion criteria to include any adult resident of the UAE. The exclusion criteria were all non-Arabic and non-English speakers. Participants were consecutively approached and asked to fill out the questionnaire.

### Tools of data collection

A self-administered questionnaire was given to the participants, along with a consent form. Face validity of the questionnaire was reviewed by one of the supervisors who is a biostatical expert, and a pilot study was done on a subset of the sample along with the analysis. The questionnaire included closed-ended multiple choice questions categorised into sections comprising:

1. Demographic data of the participant
2. History of the disease
3. Knowledge of kidney stones
4. Practices
5. Knowledge regarding renal stones prevention
6. Knowledge regarding renal stones management

### Statistical analysis

The data were coded and analysed using SPSS 25 (Statistical Package for Social Sciences). Chi-Square and t-test were used to determine correlations. Risk estimates with odds ratio (OR) were used to estimate how strongly a predictor is associated with kidney stones. Additionally, the Kruskal–Wallis test was used in the analysis. Bar and pie charts were created using MS Excel to aid in visualising the quantitative analysis of knowledge of risk factors, diagnosis methods, and complications. A p-value less than 0.5 was considered statistically significant.

## Results

This study involved the collection of data from a total of 515 participants. The age groups and percentages of the participants are indicated in Figure 1a. The distribution of the participants across both genders was approximately equal (females, 51.7% and males, 48.2%). The educational status varied across the sample, with bachelor's degree accounting for the highest percentage as indicated in Figure 1b.

**Figure 1a:**
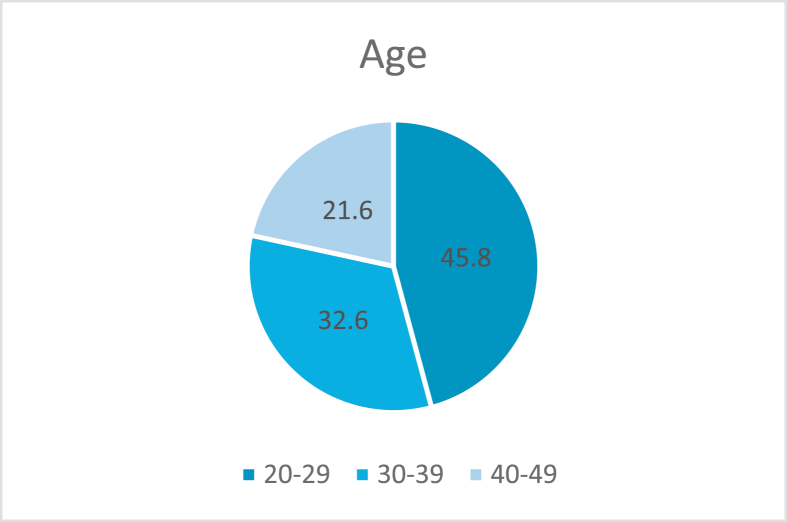
Distribution of participants by age (n = 515).

**Figure 1b:**
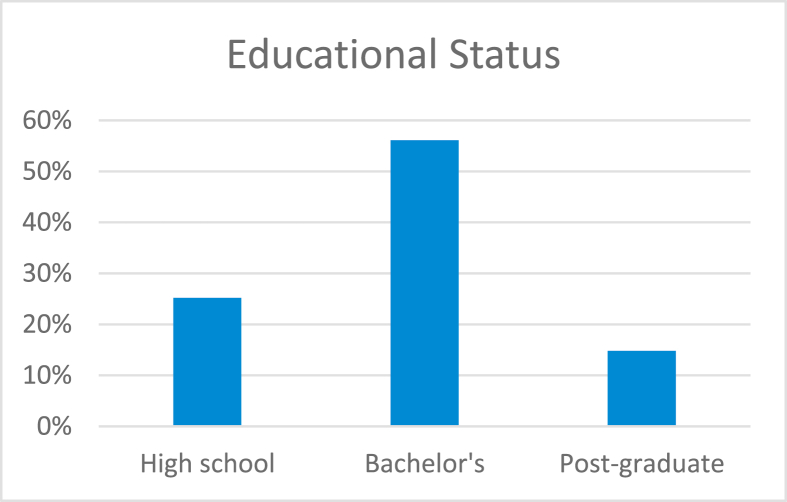
Distribution of participants by educational status (n = 515).

There was a lack of information to identify bowel condition and family history as important risk factors, as only 24.3% and 29.7%, respectively, identified it correctly. The participants’ responses were recorded and presented in Table 1. Majority of the sample (82.5%) identified the complications of kidney stones correctly. Only 6.2% incorrectly considered stool analysis as a method of investigation—a very small percentage indicating a good background on the methods of investigation of kidney stones. As shown in Table 2, participants exhibited poor level of knowledge regarding preventative foods; 80% assumed that vegetables prevent kidney stones, which in reality, is not true. The same applies for dark chocolate (27.2%) and spinach (56.5%).

**Table 1:** Responses to knowledge items regarding the formation of urinary stones.

Knowledge items	Affirmative Response (in percent)
The chances of stone formation are more in men than women	32.8
Kidney stones reoccur after some years	65.8
Climate has an effect on kidney stone	42.9
Stones can develop in kidney, ureter, and urinary bladder	80.4
Drinking more fluid will cause stone formation	7.4
Stones can damage the kidney	77.9
Urinary tract infection increases the chance of having stones	56.1
Certain bowel conditions that cause diarrhoea can raise the risk of forming kidney stones	24.3
Obesity, sitting for a long time, and being inactive increase the chances of stone formation	55
Increasing Calcium and Uric Acid in the blood will increase the chance of stone formation	57.1
Dietary modifications are not needed to prevent stones	12.4
Hormonal imbalance or gout is associated with stone formation	32.2
Kidney stones can be passed on in the family	29.7
Surgery is the only solution for treating kidney stones	13.4
Stones can be dissolved with medicines	74
Use of calcium supplements increases the risk of stone formation	41.2
Use of antacids increases the risk of stone formation	19
Use of diuretics increases the risk of stone formation	21.9
Stones up to 5 mm in size can be treated by medications	36.5
Stones more than 5 mm in size need surgery	45.4
Untreated stones can lead to kidney failure	65.4
A person with kidney stone(s) should go for life-time follow-up with regular visits to the doctor	45.8

The following statements were used to assess knowledge amongst participants (n = 515). Percentages correspond to participants who deemed these statements correct.

**Table 2:** Response to food items regarding the formation of urinary stones.

Food item	Affirmative response in percent
Water	92.4
Vegetables	80
Spinach	56.5
Citrus fruits and juices	56.1
Dark chocolate	27.2
Eggs	25.8
Nuts	24.9
Tea	16.5
Meat	10.5
Coffee	7.6
Fatty foods	4.7
Salty foods	4.5

Participants (n = 515) were asked to choose which of the following items were likely to prevent the formation of kidney stones. The likelihood, as chosen by the participants, of each food item to prevent stones, is indicated in the corresponding percentage.

A family history of kidney stones increases the risk of future stone development by 2.27 times (P-value 0.008). After comparing and correlating the results, it was found that the presence of a family history of kidney stones, which was observed in 43.1% of the population, increases their knowledge regarding the risk factors. Moreover, a prior history of kidney stones does not affect the knowledge and awareness among these participants.

The study participants lacked knowledge regarding appropriate water intake (46% had sufficient knowledge), followed by diet factors (only 50.1%) and risk factors of kidney stones (51.6%). All of these factors were combined to determine the total knowledge score, which represents the overall knowledge of the participants regarding each of the individual factors concerning kidney stones (signs and symptoms, diagnosis, complications, risk factors, appropriate water intake and dietary factors). The final mean of the total knowledge score was 56.4%, which surprisingly indicates a poor level of knowledge in the sample. In addition, 53.4% of the study population are unaware of the consequences of kidney stones. There was no difference in the knowledge scores between males and females. Nevertheless, results showed that there is no difference in the knowledge level between participants in the age groups of 20–29 and 30–39 years. However, people in the age group of 40–49 years have a significantly higher knowledge level compared to the other groups. Similar tests were performed to determine the mean difference between knowledge scores according to different educational levels; the results indicate that respondents with higher educational levels do not necessarily have higher knowledge.

Participants taking the survey were asked to identify the methods of management for kidney stones. The variables mentioned in Table 3 indicate the list of options people were presented with, which include surgery and medications used to dissolve the stone along with follow up. The results indicate that the survey respondents are evidently knowledgeable about surgical interventions not being a direct method of management; meanwhile, 63.5% were not aware that stones smaller than 5 mm are not to be referred for invasive management. Additionally, 54.6% did not know that stones larger than 5 mm are eligible for management. Finally, only 34.6% of participants did not know that if kidney stones are left untreated, they cause renal failure.

**Table 3:** Methods of disease management.

Methods of disease management	Percentage of incorrect answers
Stones must be < 5 mm to be eligible for management	63.5%
Stones must be > 5 mm to be eligible for management	54.6%
Untreated stones caused kidney failure	34.6%
Dissolution by medication	26%
Surgery	13.4%

This table shows the percentage of participants (n = 515) who identified the following management methods incorrectly.

## Discussion

This study shows that our cohort of participants have average level of knowledge about kidney stones, particularly regarding diet-related risk factors. However, the cohort shows sound knowledge and understanding about symptomatic and diagnostic factors of kidney stones. The history of kidney stones and higher educational status do not correlate with better knowledge. These findings reflect the importance of awareness of risk factors and symptomatic manifestations of kidney stones, which will enable the general population to modify lifestyles to mitigate the risk of such a common health care problem.

Interestingly, kidney stones constitute a fundamental hallmark in urological conditions that equally affect both genders. It is regarded as a multifactorial problem, influenced by family history, age, sex, diet, weather, and lifestyle.^14^^,^^15^ Metabolic Syndrome, a common disorder in the UAE, accounting for about 40.1% of the population, was found to be a key factor in the development of kidney stones. This disorder necessitates several lifestyle modifications including attention to hydration and adequate intake of minerals and essential nutritional elements.^16^, ^17^, ^18^

Determining the community's awareness and disposition about kidney stones disease, as well as its treatment and management, helps to resolve issues that might reduce its occurrence. Different environmental factors as well as dietary intake significantly contribute to the occurrence of the stones and the resultant pathological damage, which can be easily predicted by factors such as inadequate water intake and hot weather.^19^^,^^20^ In addition, most people in the UAE reach out for chilled bottles of soft drinks or consume tea and coffee instead of drinking plain and hydrating water. An informal survey has revealed that more people have green tea or a cola when they should had water instead. This obviously correlates with our topic since the biggest risk factor of kidney stones is dehydration.^19^ Historically, the Gulf countries display an increased incidence of kidney stones due to socio-economic, environmental, and nutritional factors such as high intake of oxalate-containing foods, low intakes of calcium-containing foods, and the region's dry climate. These factors eventually lead to dehydration, as it significantly increases the risk of development of renal stones. This highlights the importance of assessing the population's knowledge levels, as an improved understanding about these factors could help reduce morbidity related to kidney stones, specifically through early detection and management.^21^

In the study conducted by Baatiah et al. in KSA in 2017, the investigators reported the prevalence of urolithiasis in the community to be as high as 11.2%,^21^ The study further states that 37.7% respondents had low levels of awareness; 35.3%, moderate; and around 0.6%, high levels of awareness. It also reiterates the significance of improving the knowledge base of the general population about kidney stones by conducting nationwide public awareness campaigns among all age groups of the community.

Studies have shown that a great majority of primary care physicians were aware of suitable preventive measures against recurring kidney stones. However, this information does not appear to be effectively implemented in clinical practice. Very few articles have focused on the awareness and understanding of individuals in our cohort. Limited data about the knowledge and understanding of the general population regarding renal stones is available in the UAE.^22^^,^^23^ It is worth noting that the presence of modifiable and unmodifiable factors can potentially lead to malignant transformations in different human organs, such as low vitamin D levels and breast cancer,^24^ family history for malignant melanoma,^25^ high animal fat consumption for colorectal cancer,^26^ and cholesterolosis for gallbladder carcinoma.^27^ Similarly, long-standing stones in the urinary tract have been shown to be potentially carcinogenic and can lead to renal cell carcinoma and transitional cell carcinoma of the kidneys.^28^

It is important to raise people's awareness of whether they have any pain that may be linked to the development of kidney stones and to clarify how to distinguish between them and any other diseases. It is also important to guide people towards a better and healthier lifestyle, especially in terms of water consumption (because people in the UAE are in a hot climate region), eating a healthy diet, tracking weight, and physical activity that will help reduce certain cases of formation of kidney stones and prevent further complications.^29^ The significance of this research is that it seeks to clarify the proportion of our study sample that is aware of the risk factors of kidney stones, so that, if necessary, campaigns can be carried out to raise awareness of prevention. Consequently, the knowledge levels among the population will grow to encounter these risk factors or even the disease directly, and the education and expertise of the current population will influence the understanding of the next generation.

## Limitations

The study used a non-probability convenience sampling method which affects the generalisability of the results. The information was acquired through self-reporting which is vulnerable to recall bias. Some of the cited articles used in this study are outdated and were used due to the lack of recent papers in the region on this topic.

## Conclusion

Participants in general had varied responses to the different aspects of knowledge regarding kidneys stones. The findings indicate that approximately half of the respondents are somewhat aware of certain aspects regarding kidney stones, including complications, diagnosis, and management. Individuals who have a family history of kidneys stones had a compellingly higher level of knowledge about kidney stones compared to the other group. In addition, participants in the age group of 40–49 years had a significantly higher knowledge level compared to the other groups. However, participants with a higher education level did not necessarily have greater knowledge than the rest of the population, which was quite enlightening.

## Recommendation

A public health intervention aimed at correcting misconceptions and boosting preventative measures is recommended. A detailed inquiry of how and why the community incorporates certain behaviours that prevent urinary stones is worth investigating. A large-scale study that incorporates these points with a larger number of participants is recommended. Moreover, this study revealed the lack of knowledge in some disease aspects, which highlights the need for public health awareness regarding this disease.

## Source of funding

This study did not receive any specific grant from funding agencies in public, commercial, or not-for-profit sectors.

## Conflict of interest

The authors have no conflict of interest to declare.

## Ethical approval

Ethical approval was obtained from the local Research Ethics Committee on 17 February 2019, Reference number: REC-18-12-06-04-S.

## Authors' contribution

SF conceptualised the idea. DA prepared the design and research instrument. AH and HS performed data collection and processing. HS carried out data analysis. NM and EA interpreted research data. All authors have critically reviewed and approved the final draft and are responsible for the content and similarity index of the manuscript.
